# Weakly acidic pH reduces inflammatory cytokine expression in airway epithelial cells

**DOI:** 10.1186/s12931-016-0399-3

**Published:** 2016-07-15

**Authors:** A. P. Hackett, R. E. Trinick, K. Rose, B. F. Flanagan, P. S. McNamara

**Affiliations:** Department of Women’s and Children’s Health, Institute of Translational Medicine, University of Liverpool, Liverpool, UK; Alder Hey Children’s Hospital NHS Foundation Trust, Eaton Rd, Liverpool, UK

**Keywords:** Reflux-aspiration, Airway epithelial cells, Neurodisability, BAL, Interleukin-6, Interleukin-8, Lipopolysaccharide, Inflammation

## Abstract

**Background:**

Aspiration lung disease (ALD) is a common cause of respiratory morbidity in children and adults with severe neurodisability (sND). Recent studies suggest that chronic microaspiration of gastric contents is associated with mild rather than low, airway acidification. We investigated inflammatory responses to infection by airway epithelial cells (AECs) exposed to weakly acidic media.

**Methods:**

Using pH measurements from children with sND at high risk of ALD as a guide, we incubated AECs in weakly acidic (pH5.5–7.4) media alone; in combination with lipopolysaccharide (LPS); or prior to LPS stimulation at normal pH. Interleukin (IL) -6 and IL-8 expression were measured.

**Results:**

IL-6/8 expression in AECs simultaneously exposed to weakly acidic media and LPS for 4 h was reduced with no effect on cell viability. Pre-incubation of AECs at weakly acidic pH also reduced subsequent LPS-induced cytokine expression. Suppression of inflammation was greatest at lower pHs (pH 5.5–6.0) for prolonged periods (16/24 h), but this also adversely affected cell viability.

**Conclusion:**

AEC inflammatory responses to bacterial stimuli is markedly reduced in a mildly acidic environment.

## Background

Reflux-aspiration, the regurgitation of gastric material up the gastro-oesophageal tract and its subsequent penetration into the lower airways, is associated with respiratory disorders such as idiopathic pulmonary fibrosis, chronic obstructive pulmonary disorder and bronchiolitis obliterans syndrome in lung transplant patients [1–4]. It is also a common cause of respiratory morbidity and mortality in children and adults with severe neurodisability (sND) [5–7].

Aspiration episodes can occur acutely as bolus events leading to acute lung injury and pneumonia, or chronically, through repeated aspiration of small volumes of gastric aspirate leading to chronic respiratory symptoms [8]. There is also emerging consensus that “silent reflux” with chronic microaspiration of aerosolised, non-acidic or weakly acidic, gastric material is a clinical entity [9, 10]. Supporting evidence comes from oesophageal and tracheal pH measurements in patients with suspected reflux-aspiration and chronic respiratory disease, in whom tracheal pH rarely drops below pH 5.0 even with reflux episodes [7, 11–14]. It is speculated that this relatively mild airway acidification leads to infection, persistent inflammation, and irreversible lung damage over time. In bronchoalveolar lavage fluid (BAL) from children with sND at high risk of chronic microaspiration, we have recently shown bacterial airway colonisation with oral commensals associated with elevated levels of IL-8 and marked airway neutrophilia [15]. In vitro and in vivo studies have previously shown that airway epithelial cells (AECs) exposed to acid produce pro-inflammatory and chemoattractant cytokines leading to leukocyte infiltration/activation in the airways, but most of these studies involved the administration of low pH (i.e. pH < 3.0) boluses [16–20]. As such, they are unlikely to reflect the scenario in those patients described above with chronic reflux-aspiration.

In this study, we have documented BAL pH in a group of children at high risk of reflux-aspiration. We then investigated the effects of mild acidification (pH range 7.4 – 5.5) on inflammatory responses of airway epithelial cells (AECs). These studies have led us to explore the consequences of acidification in the context of infection, through exposure of AECs to mild acidification followed by challenge with lipopolysaccharide (LPS), a major component of the outer membrane of Gram-negative bacteria.

## Methods

### Patients

BAL was collected from children admitted to Alder Hey Children’s Hospital in Liverpool, UK between October 2009 and September 2011 as part of a larger study into airway pathology in children with sND. The Liverpool Paediatric Research Ethics Committee reviewed and approved the study and consent procedure (REC Reference number: 09/H1002/58). Informed written parental consent was obtained. Children over the age of 2 years with central ND (not neuromuscular disease) who were non-ambulant (Gross Motor Function Classification IV-V) were recruited at a time of respiratory stability when admitted for elective surgical procedures, or at a time of respiratory deterioration whilst ventilated on the paediatric intensive care unit (PICU).

### Sample collection and processing

Non-bronchoscopic BAL samples were collected according to European Respiratory Society (ERS) guidelines following induction of anaesthesia [21]. Within 15 min of collection, BAL sample pH was measured using a pH meter (Philips PW 9418, Netherlands), calibrated prior to each reading.

### Cell culture and experimental conditions

BEAS-2B bronchial epithelial cells were used as a model of healthy airway epithelial cells and grown in BEGM media (Lonza, Belgium) on flasks coated with 1 % PureCol® collagen (Nutacon, Netherlands) in HEPES buffered saline solution under humidified conditions at 37 °C and 5 % CO_2_. All experiments were carried out between passage 3–5. The same passage of cells was used to test each experimental condition. Each biological replicate was carried out on the subsequent passage. Cells were seeded at 3×10^3^ or 2×10^4^ cells per well in 96-well and 24-well plates respectively, and grown to 70 % confluence having being fed 24 h before each experiment. Cells were incubated for 4, 8, 16 or 24 h at varying pH. Media pH was adjusted to pH7, pH6.5, pH6 and pH5.5 through the addition of 1 M HCl. Cells were stimulated with *Escherichia coli* LPS (Sigma, UK) at 5 μg/ml for 4 or 16 h in acidic media or for 24 h in normal pH media (pH7.4). For some experiments, cells were treated with the intracellular protein transport inhibitor, Brefeldin A (eBioscience, UK), for 1 h before addition of LPS at pH7.4. This acts as a positive control for cytosolic protein retention following cell activation. Images of cells were taken using a phase-contrast microscope with a DFC420 camera (Leica, Germany).

### Preparation of whole cell lysates

At the end of each time point, media was removed from cells and centrifuged. Cell-free supernatant was stored for future analysis at −30 °C. Cells were twice washed with ice cold sterile PBS and lysed using Cytobuster™ Protein Extraction Reagent (Merck Millipore, Germany) following the manufacturer’s instructions. Whole cell lysate was stored at − 30 °C for future analysis.

### Cytokine mRNA expression

IL-6 and IL-8 was measured by quantitative real-time PCR (qPCR). RNA was extracted from cells using the RNeasy MiniKit (Qiagen, Netherlands) following the manufacturer’s instructions. Reverse transcription was performed using a High Capacity cDNA Reverse Transcription Kit (Applied Biosystems, UK) and qPCR was performed using TaqMan primer probe assays: IL-6, Hs00985639_m1; IL-8, Hs00174103_m1; L32, Hs00388301_m1; β-actin, Hs99999903_m1 (Life Technologies, USA). Ribosomal protein L32 and β-actin were used as internal standards [22, 23]. Expression was measured in duplicate and was calculated using the comparative C_T_ method [24].

### Cytokine protein measurement

Intracellular and extracellular IL-6 and IL-8 protein expression was quantified in whole cell lysate and culture supernatant by ELISA (R&D Systems, USA). Intracellular cytokine concentration was normalised to the total protein concentration of whole cell lysate as measured by BCA protein assay (Pierce, UK). Cytokine and total protein concentrations were measured in duplicate. Interleukin protein stability at pH ≥ 5.5 was confirmed by spike retrieval assay.

### Cell viability measurement

Cell viability was measured using an MTT assay (Life Technologies, USA). MTT is a tetrazolium dye, taken up by live cells and therein reduced to a purple insoluble product. This reaction can be quantified and used as a measure of metabolic activity and an indicator of cell viability. Following incubation under experimental conditions, cell media was replaced with fresh BEGM (pH7.4). MTT dye solution was added to the normal pH media in each well for 4 h at 37 °C and 5 % CO_2_. All but 25 μl media was then removed and cells incubated for 10 min at 37 °C with 50 μl DMSO to solubilize the cytosolic formazan product. Each well was mixed thoroughly by pipetting and the plate was read at 540 nm. Cell viability was expressed as a percentage of the control OD value. Viability was measured in triplicate on a 96-well plate.

### Statistical analysis

StatsDirect 2.7.9 (StatsDirect Ltd, UK) was used for statistical analysis of experimental data. BAL pH was analysed by Mann-Whitney test. All qPCR data was analysed by Kruskal-Wallis one-way analysis of variance followed by Conover-Inman pairwise comparison. ELISA data was analysed by Kruskal-Wallis one-way analysis of variance followed by Conover-Inman pairwise comparison. MTT assay data was analysed by one-way ANOVA followed by Dunnett’s multiple comparison test. Values are presented as mean ± SEM. Statistical significance was defined as *p* < 0.05.

## Results

### BAL pH from children with sND

BAL pH from elective-ND patients (*n* = 8) was generally acidic (median [range] pH 6.5 [5.5–7.2]). In contrast, BAL pH from PICU-ND patients (*n* = 9), while not significantly different (*p* = 0.061), was more frequently alkaline (pH 7.3 [5.0–7.7]) (Table 1). There was extensive variability in BAL pH in both groups.

**Table 1:** pH range observed in patient BAL - pH of BAL collected from elective ND patients and PICU-ND patients

	Elective ND	PICU ND
pH of BAL	5.5	6.9
	6.3	7.3
	6.3	7.7
	6.7	7.3
	6.1	7.4
	6.9	7.4
	6.7	5
	7.2	6
		7.3
Number of Patients	8	9
Maximum pH	7.2	7.7
Minimum pH	5.5	5
Median pH	6.5	7.3
Standard Error	0.19	0.29

pH of BAL collected from elective ND patients and PICU-ND patients

### Expression of inflammatory cytokines by AECs in response to a weakly acidic environment

**Fig. 1:**
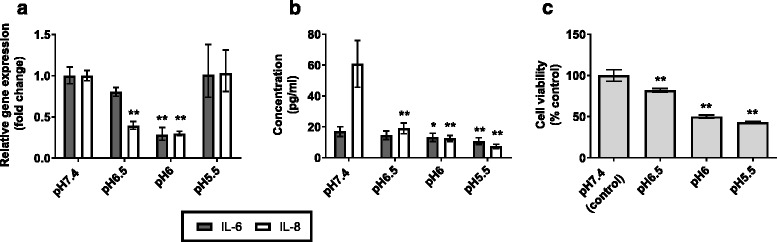
Epithelial cell response to 24 h incubation in weakly acidic media. AECs were incubated in media adjusted to pH6.5 – pH5.5 with HCl for 24 h (*n* = 3). Normal, unadjusted BEGM is pH7.4; this was used as a control to show basal IL-6 and IL-8 expression. **a** Expression of IL-6 and IL-8 mRNA was measured by qPCR. **b** Corresponding protein secretion was measured in pg/ml by ELISA. **c** Cell viability was measured by MTT assay. Values presented are mean ± SEM *p < 0.05, **p < 0.01

To determine the inflammatory effect of prolonged, mild acidification of AEC extracellular environment, we measured expression of two key pro-inflammatory cytokines IL-6 and IL-8 by BEAS-2B bronchial epithelial cells in response to pH-adjusted media for 24 h (Fig. 1).

Mean IL-8 mRNA expression was significantly reduced from control (pH7.4) in those cells incubated at pH6.5 (*p* < 0.001) and pH6 (*p* < 0.001). IL-6 mRNA expression was also reduced in cells incubated at pH6 (*p* < 0.001) (Fig. 1a). A corresponding significant reduction was observed in the concentration of IL-6 and IL-8 secreted protein at pH6.5 (IL-8, *p* = 0.002) and pH6 (IL-6, *p* = 0.042; IL-8, *p* < 0.001) (Fig. 1b). At pH5.5, mRNA expression of both IL-6 and IL-8 was no different from cells incubated at pH7.4, however, secreted protein expression of these cytokines was significantly reduced (*p* < 0.001). Prolonged exposure of AECs to weakly acidic media for 24 h did reduce cell viability. At pH6.5, mean cell viability was 82 % of pH7.4 control, and further reduced to 50 and 43 % at pH6 and pH5.5 respectively (Fig. 1c).

### Response of AECs to LPS challenge in a weakly acidic environment

It has previously been shown that cytokine retention by macrophages in weakly acidic conditions leads to attenuated LPS-induced TNF-α secretion [25–29]. To determine if this occurs in AECs, we investigated whether weakly acid conditions reduce/inhibit inflammatory responses to exogenous stimuli. Thus, AECs were incubated at pH7.4–5.5 with LPS for relatively short (4 h) and more prolonged (16 h) periods of time in an attempt to replicate changes in lung pH in response to reflux-aspiration. Brefeldin A is a fungal lactone which inhibits protein secretion by disrupting transport of proteins between the endoplasmic reticulum and the Golgi apparatus [30]. It was used with LPS as a positive control for cytosolic protein retention.

**Fig. 2:**
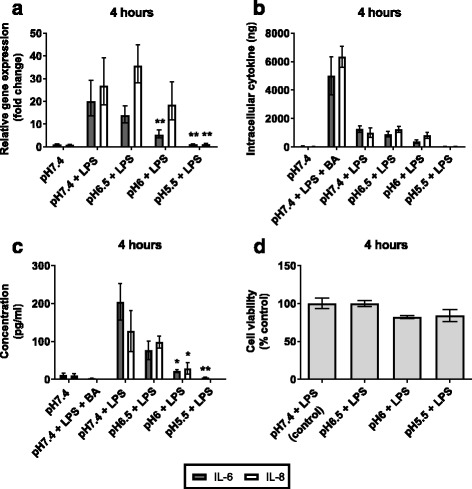
Inflammatory cytokine expression in AECs stimulated with LPS in weakly acidic media for 4 h. AECs were stimulated with 5 μg/ml LPS in media adjusted to pH6.5 – pH5.5 with HCl for 4 h (*n* = 3). Normal, unadjusted BEGM is pH7.4; this was used as a control to show basal IL-6 and IL-8 expression. LPS stimulation at pH7.4 was used as a control for normal AEC response to LPS. Brefeldin A (BA) was used with LPS as a positive control for intracellular protein retention. **a** IL-6 and IL-8 mRNA expression was measured by qPCR. Fold change is related to pH7.4 control. **b** Cytosolic IL-6 and IL-8 protein was measured in pg/ml by ELISA of whole cell lysates and was normalised to total protein concentration, measured in μg/ml by BCA assay. Cytosolic cytokine was calculated in ng. **c** Secretion of IL-6 and IL-8 proteins was measured in pg/ml by ELISA of cell supernatants. **d** Cell viability was measured by MTT assay. (*n* = 3) All test pH conditions were compared to pH7.4 + LPS for statistical analysis. Values presented are mean ± SEM *p < 0.05, **p < 0.01

**Fig. 3:**
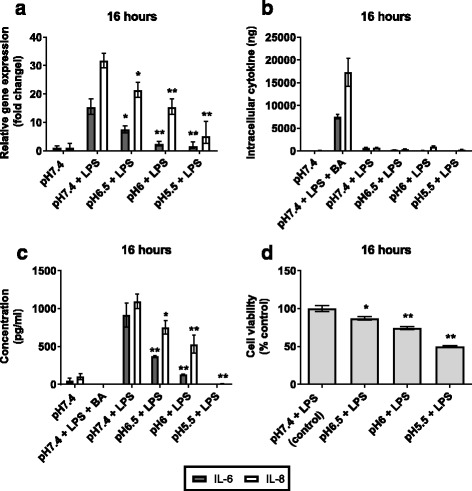
Inflammatory cytokine expression in AECs stimulated with LPS in weakly acidic media for 16 h. AECs were stimulated with 5 μg/ml LPS in media adjusted to pH6.5 – pH5.5 with HCl for 16 h (*n* = 3). Normal, unadjusted BEGM is pH7.4; this was used as a control to show basal IL-6 and IL-8 expression. LPS stimulation at pH7.4 was used as a control for normal AEC response to LPS. Brefeldin A (BA) was used with LPS as a positive control for intracellular protein retention. **a** IL-6 and IL-8 mRNA expression was measured by qPCR. Fold change is related to pH7.4 control. **b** Cytosolic IL-6 and IL-8 protein was measured in pg/ml by ELISA of whole cell lysates and was normalised to total protein concentration, measured in μg/ml by BCA assay. Cytosolic cytokine was calculated in ng. **c** Secretion of IL-6 and IL-8 proteins was measured in pg/ml by ELISA of cell supernatants. **d** Cell viability was measured by MTT assay. (*n* = 3) All test pH conditions were compared to pH7.4 + LPS for statistical analysis. Values presented are mean ± SEM *p < 0.05, **p < 0.01

At 4 h, LPS-induced IL-6 mRNA expression was reduced at pH6 and pH5.5. In contrast, LPS-induced IL-8 expression was only significantly reduced at pH5.5 (IL-8, *p* = 0.005) (Fig. 2a). This effect was more pronounced at 16 h, with significant reduction in both IL-6 and IL-8 mRNA expression following LPS stimulation observed at pH6.5 (IL-6, *p* = 0.044; IL-8, *p* = 0.027) and below (pH6: IL-6, *p* < 0.001; IL-8, *p* = 0.002, pH5.5: IL-6, *p* < 0.001; IL-8, *p* < 0.001) (Fig. 3a). No increase in cytosolic protein expression was detected from cells incubated in weakly acidic media, in marked contrast to the increase observed in Brefeldin A treated cells (Figs. 2b and 3b). Secreted interleukin protein expression was reduced in a corresponding manner to mRNA expression (Figs. 2c and 3c).

Cell viability at 4 h, as measured by MTT assay, was not significantly different at pH ≤6.5 with LPS, compared to pH 7.4 with LPS (Fig. 2d). At 16 h, viability was reduced in cells exposed to LPS at pH6 and pH5.5 (Fig. 3d).

### Response of AECs to LPS challenge at pH7.4 following pre-incubation in a weakly acidic environment

**Fig. 4:**
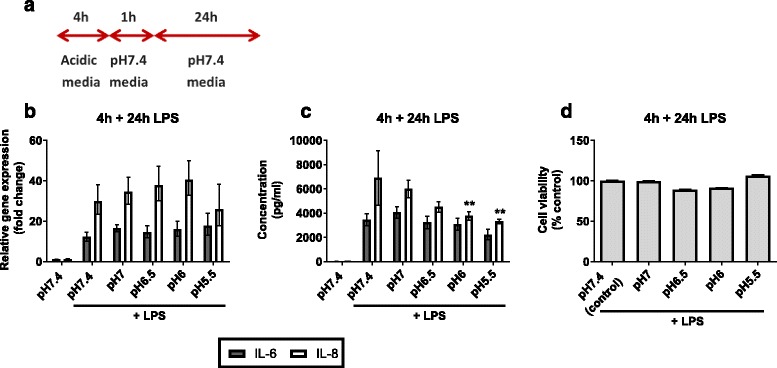
Inflammatory cytokine expression in AECs “shock treated” for 4 h prior to 24 h LPS stimulation. AECs were incubated in weakly acidic media for 4 h prior to stimulation with LPS in pH7.4 media for 24 h. Expression of inflammatory cytokines IL-6 and IL-8 was measured at the 24 h time point. **a** Schematic representation of experimental plan. **b** IL-6 and IL-8 mRNA expression was measured by qPCR. **c** Secretion of IL-6 and IL-8 protein was measured by ELISA of cell supernatants **d** Cell viability measured by MTT assay. (*n* = 3) All test pH conditions were compared to pH7.4 + LPS for statistical analysis. Values presented are mean ± SEM *p < 0.05, **p < 0.01

**Fig. 5:**
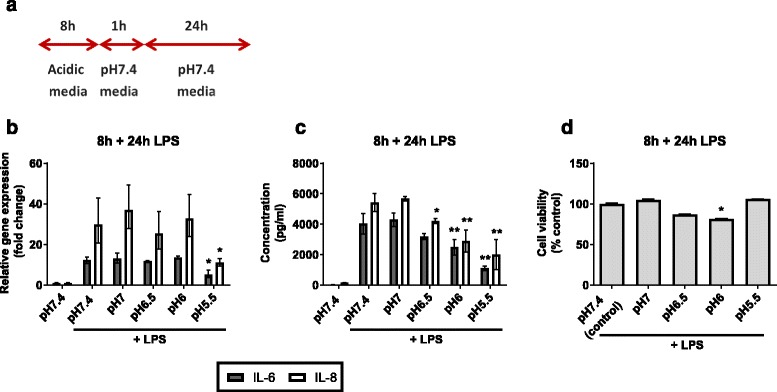
Inflammatory cytokine expression in AECs “shock treated” for 8 h prior to 24 h LPS stimulation. AECs were incubated in weakly acidic media for 8 h prior to stimulation with LPS in pH7.4 media for 24 h. Expression of inflammatory cytokines IL-6 and IL-8 was measured at the 24 h time point. **a** Schematic representation of experimental plan. **b** IL-6 and IL-8 mRNA expression was measured by qPCR. **c** Secretion of IL-6 and IL-8 protein was measured by ELISA of cell supernatants. **d** Cell viability measured by MTT assay. (*n* = 3) All test pH conditions were compared to pH7.4 + LPS for statistical analysis. Values presented are mean ± SEM *p < 0.05, **p < 0.01

To determine whether prior exposure to mild acidification reduces subsequent inflammatory responses to bacterial infection, AECs were pre-incubated in acidic media for 4 or 8 h and then stimulated with LPS at pH7.4 for 24 h (Figs. 4a and 5a). At the end of the experiment, IL-6 and IL-8 mRNA and protein expression were analysed, and an MTT assay performed to assess cell viability.

LPS-induced IL-6 and IL-8 mRNA expression in cells pre-incubated at pH6.5 or pH6 for both time points was no different than in those cells pre-incubated at pH7.4 (Figs. 4b and 5b). However, mRNA expression did decrease in those cells incubated in pH5.5 media for 8 h prior to LPS stimulation (IL-6, *p* = 0.025; IL-8, *p* = 0.043).

Secreted IL-6 and IL-8 protein expression differed in their patterns of response to LPS challenge following acidification. Secreted IL-8 from cells pre-incubated at pH6 (*p* = 0.009) and pH5.5 (*p* < 0.001) for 4 h was less than from cells at pH7.4, whereas secreted IL-6 protein expression remained unchanged (Fig. 4c). Cells pre-incubated at weakly acidic pH for 8 h showed a decrease in LPS-induced IL-8 protein expression (*p* < 0.05) (Fig. 5c). A significant decrease in IL-6 expression was observed in those cells pre-incubated at pH6 and pH5.5 (*p* < 0.001).

**Fig. 6:**
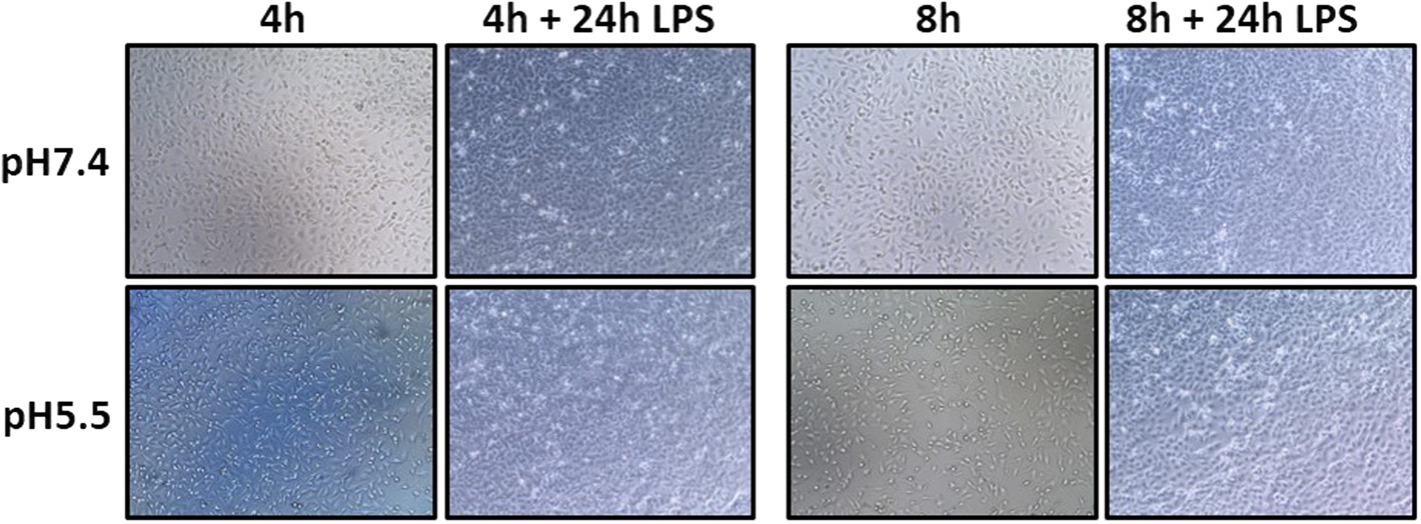
Light microscope images of cells were taken after 4 h or 8 h pre-incubation in normal (pH7.4) or weakly acidic (pH5.5) media. Images of cells from the same wells were taken after subsequent incubation with LPS at pH7.4 for 24 h

No significant change in cell viability was observed for those cells pre-incubated for 4 h at pH7.0–5.5 (Fig. 4d). Cell viability remained similarly stable following 8 h pre-incubation with significant reduction to 81 % only seen at pH6. (Fig. 5d). Microscope images were taken following pre-incubation in acidic media and at the end of the experiment (Fig. 6). Cells incubated in control (pH7.4) media for the duration of the experiment display the typical cobblestone morphology associated with BEAS-2B cells and ongoing proliferation is observed following stimulation with LPS for 24 h (Fig. 6, upper panel). Following pre-incubation at pH5.5 for 4 h some cells have detached from the surface of the well. Those cells which remain attached appear generally more dispersed and cell-cell contact is reduced. These features are more marked following 8 h pre-incubation. At the end of each pre-incubation step media was replaced with pH7.4 media prior to LPS stimulation – any detached cells were removed by this process. Images taken at the end of the experiment show that cells pre-incubated in pH5.5 media have proliferated to the same confluence as cells pre-incubated in pH7.4 media (Fig. 6, lower panel).

## Discussion

Chronic microaspiration of gastric reflux has been implicated in the pathogenesis of multiple respiratory diseases, yet our understanding of how aerosolized refluxate affects the airways remains limited. In this manuscript we report that BAL pH ranges varied between pH5.0–7.7 in individuals at high risk of aspiration. Consequently, we investigated inflammatory responses of AECs to a weakly acidic environment. We found that prolonged exposure resulted in both cytotoxicity but did not induce inflammatory cytokine expression. However, exposure for shorter periods (4 h), while not significantly affecting cytotoxicity, did blunt epithelial responses to LPS, an effect not associated with increased intracellular cytokine retention. Suppression of epithelial inflammatory responses without significant cytotoxicity continued to be seen when we “shock treated” cells with acid prior to stimulation with LPS at normal pH.

We based our in vitro experimental design on evidence from combined oesophageal and tracheal pH monitoring studies in individuals with gastro-oesophageal reflux-associated asthma, stroke and cystic fibrosis which rarely report drops in pH below pH5.0 [7, 11, 14]. Studying a pH range of pH7.4–5.5 was further supported by the pH range of BAL collected from children at high risk of aspiration lung disease undergoing routine elective surgery. Furthermore, a recent study investigating reflux-aspiration in critically ill children found that the majority of reflux episodes were weakly acidic (pH4-7) [31]. Our BAL pH data implies that there is variability in the airway pH of individuals at high risk of aspiration. As there is no definitive test for reflux-aspiration [32], this variability may be explained by the fact that not all patients measured are aspirating their refluxate. We accept that in our cohort, airway acidification may not have solely been due to reflux-aspiration. Lower airway inflammation, so often seen in these children [15], is characterised by an acidic microenvironment believed to be a consequence of neutrophil infiltration and bacterial metabolism [33–36]. Of note in our study, BAL pH in those children with ND who were mechanically ventilated for a respiratory exacerbation (PICU-ND), was generally more alkaline (*p* = 0.06), a finding that probably reflects lower carbon dioxide levels in the airways of these patients. Additionally, we have previously reported that BAL from PICU-ND patients had higher protein concentrations than BAL from elective patients [15]. It is known that high concentrations of proteins, such as albumin, can buffer pH therefore it is possible that this may contribute to the higher pH of PICU-ND BAL.

Previous studies in mice have reported the effects of acid aspiration on pulmonary inflammatory cytokine secretion and neutrophil recruitment [16–20]. These studies are probably not physiologically relevant in the context of chronic microaspiration of aerosolized gastric fluid, as the observed inflammation and neutrophilia was due to acute tissue injury caused by the direct administration of very low pH solutions (pH1.25) into the airways. In contrast, we have studied prolonged exposure to a weakly acidic environment, and found that while it does cause some cell death, it also suppresses inflammatory cytokine production. This suppression may be influenced by the reduction in viable cells following 24 h incubation in weakly acidic media, although it is surprising that this cytotoxicity did not induce an inflammatory response. Given that airway epithelium is regularly exposed to a variety of inhaled exogenous stimuli, there will undoubtedly be a necessity for a degree of stimulatory tolerance. It is conceivable that AECs are relatively refractory to ‘mild’ changes in pH within their microenvironment [37], and certainly capable of recovering from such insults.

Repeated respiratory tract infections are common in individuals who aspirate [6], with bronchiectasis a common end stage pathology [38]. Weakly acidic conditions can blunt inflammatory responses to infection exhibited by macrophages [25, 26, 28, 36, 39]. Furthermore, a reduction in airway surface liquid pH in the CF pig model reduces bacterial killing, a finding attributed to loss of antimicrobial peptide activity [40]. As AECs play an important role in regulating local immunity through secretion of chemoattractants such as IL-8 in response to infection [37], we hypothesized that weakly acidic conditions might reduce these responses. Consequently, we investigated whether AEC ability to express inflammatory/chemotactic proteins in response to LPS stimulation was altered in an acidic environment. LPS-induced cytokine expression was reduced when AECs were incubated for 4 h at weakly acidic pH, with cells maintaining viability. Expression was further reduced on 16 h exposure to low pH, but was associated with a significant decrease in cell viability.

There is little information in the published literature about duration of microaspiration episodes. We postulated that maximum duration of airway exposure to refluxate might occur at night-time and be up to 8 h. We therefore investigated whether discrete ‘shock treatments’ of 4 or 8-hours weakly acidic pH exposure might influence subsequent airway inflammatory responses to endotoxin. When cells were viewed by microscopy immediately following ‘shock’ treatment, some cells had detached (particularly those incubated at pH5.5), implying a degree of cell death. However, following subsequent 24-hour incubation at pH7.4 with LPS, all cultures appeared macroscopically similar to those maintained in pH7.4 media throughout, with cell viability well preserved. Again, we observed a significant reduction in LPS-induced cytokine protein production following weakly acidic shock treatment and subsequent prolonged exposure to endotoxin at normal pH. It is possible that similar exposure in individuals who micro-aspirate might compromise their response to infection through reduced cytokine production diminishing immune cell recruitment.

Interestingly, the metabolic rate of AECs “shocked” at pH5.5 and measured by MTT assay, was higher than cells maintained at higher pH (including those at pH7.4). Given that this pH obviously causes some cellular injury/detachment, it may be that those cells that survive this “shock” treatment increase their rate of proliferation in an effort to heal the monolayer. It is possible that this type of repeated injury could occur in individuals that chronically aspirate, resulting in loss of epithelial tissue and potentially leading to airway remodelling, a finding commonly observed in patients suspected of reflux-aspiration [4].

Whilst there are limitations to this primarily in vitro study, the experimental design we used was more physiologically relevant in the context of chronic microaspiration of gastric refluxate than previously published work examining neat administration of hydrochloric acid. We accept that our in vitro monolayer culture model has a number of limitations including the lack of cellular differentiation and that our proxy aspirate lacked other important components of gastric juice such as pepsin that could also affect AEC function. Nevertheless, this work is novel and should be informative for future investigations into this much under-researched respiratory pathology.

## Conclusions

In summary, this is the first published study to examine the effects of mild acidification on inflammatory responses of AECs. We have shown that AECs are surprisingly tolerant to mild acidification but that this environmental alteration may affect their ability to induce an adequate inflammatory response to bacterial stimulus.

## Abbreviations

AEC, airway epithelial cell; BA, Brefeldin A; BAL, bronchoalveolar lavage fluid; BCA, bicinchoninic acid; BEGM, bronchial epithelial cell growth medium; DMSO, dimethyl sulfoxide; HCl, hydrochloric acid; IL-6, interleukin 6; IL-8, interleukin 8; LPS, lipopolysaccharide; MTT, 3-(4,5-dimethylthiazol-2-yl)-2,5-diphenyltetrazolium bromide; qPCR, quantitative real-time PCR; sND, severe neurodisability; TNF-α, tumour necrosis factor α
